# Comparing a common clavicle maturation-based age estimation method to ordinary regression analyses with quadratic and sex-specific interaction terms in adolescents

**DOI:** 10.1038/s41598-024-52980-x

**Published:** 2024-02-02

**Authors:** Sebastian R. Reder, Isabel Fritzen, Marc A. Brockmann, Jochen Hardt, Katrin Elsner, Katja Petrowski, Monika Bjelopavlovic

**Affiliations:** 1 Department of Neuroradiology, University Medical Center, Johannes Gutenberg-University of Mainz, 55131 Mainz, Germany; 2 Department of Prosthetic Dentistry, University Medical Center of the Johannes Gutenberg-University Mainz, Augustusplatz 2, 55131 Mainz, Germany; 3 Department of Medical Psychology and Medical Sociology, University Medical Center of the Johannes Gutenberg-University Mainz, Duesbergweg 6, 55131 Mainz, Germany; 4 Institute of Legal Medicine, University Medical Center of the Johannes Gutenberg-University of Mainz, Am Pulverturm 9, 55131 Mainz, Germany

**Keywords:** Ageing, Cartilage development, Anatomy, Medical research, Physical examination, Population screening

## Abstract

Established methods of age estimation are based on correlating defined maturation stages of bony structures with tables representing the observed range of biological ages in the majority of cases. In this retrospective monocentric study in southwestern Germany, common age estimation methodology was assessed in n = 198 subjects at the age of 25 or younger by analyzing the influence of age, quadratic age, biological sex and age-sex interaction on the ossification stages of the medial epiphysis fugue. Three readers (ICC ≥ 0.81 for left/right side) evaluated routine care computed tomography images of the clavicle with a slice thickness of 1 mm. By using least square regression analyses, to determine the real biological age a quadratic function was determined corrected for the age estimated by established methods and sex (R^2^ = 0.6 each side), reducing the mean absolute error and root mean squared error in the age estimation of women (2.57 and 3.19) and men (2.57 and 3.47) to 1.54 and 1.82 for women, and 1.54 and 2.25 for men. In women, the medial clavicle epiphysis seem to fuse faster, which was particularly observable from approximately 18 years of age. Before that age, the estimation method was relatively close to the ideal correlation between assessed and real age. To conclude, the presented new method enables more precise age estimation in individuals and facilitates the determination and quantification of additional variables, quantifying their influence on the maturation of the medial clavicle epiphysis based on the established ossification stages.

## Introduction

In the field of radiology, it is customary to acquire a two-dimensional hand radiograph for age determination in individuals^1^. However, after the hand growth has reached maturity, a thin-slice CT scan of the clavicles can be conducted, allowing for staging based on appropriate reference tables and standard deviations^1^. The medial clavicular epiphysis, being the final bone to ossify during development, offers significant biological insights into an individual's age^2,3^. The original classification of ossification into stages 1–5 (Schmeling’s classification) has undergone further refinement with the incorporation of subgroups (2a-c and 3a-c according to Kellinghaus’ classification)^3–5^. Substage 3c signifies the completion of 19 years of age, while the presence of stage 4 suggests that an individual has attained 21 years of age^5^. The clavicle has been recognized as a dependable biological structure for forensic age estimation in the literature and can contribute to determining the question of legal adulthood^6,7^. Some studies have explored age estimation by combining information from both clavicle and wisdom teeth analyses, although only a few investigations have examined these structures individually using a single CT dataset^8^. A multifactorial approach aligned with AGFAD standards (“Arbeitsgemeinschaft für Forensische Altersdiagnostik”; engl.: working group for forensic age estimation in Germany) is imperative, with a population-based focus to ensure precise expert opinions. Overall, the utilization of a thin-slice CT scan of the clavicles (less equal 1 mm slice thickness) offers a reliable method for determining an individual's age and can be employed alongside other techniques for forensic age estimation^9^.

The development of sex phenotype primarily relies on hormonal and genetic influences in mammals, with environmental factors suspected to play a role affecting the hormone balance estrogenically or antiandrogenically^10–17^. However, in clinical studies, significant sex hormone-specific effects on the epidemiology, diagnosis, treatment, and prognosis of diseases have been observed repeatedly^18–26^. Regarding to the processes of ossification, epiphyseal fusion in various bones is affected by sex-specific endocrine factors, as well as the gain of total bone mass^4,12,27–31^. Interestingly, no relevant sex-specific differences in earlier stages of ossification of the medial clavicular epiphyseal fusion were observed (up to stage 3, correlated with approximately 16 years of age), whereas in females later clavicle stages appear earlier than in males (stage 4, correlated with approximately 20 years of age)^4^. These observations were supported by investigations of the relative maturation in clavicle length growth^3,5^. Around 80% of final length growth was completed in girls by the age of 9 and in boys by the age of 12^32^. Additionally, sex-specific differences in the morphology of the clavicle and the humerus have been reported^33,34^. In parallel, Akhlaghi et al. (2012) utilized morphological differences in the clavicle to reliably determine the sex of deceased individuals^35^.

Regarding the endocrine mechanisms, until the age of estimated nine years the basic growth rate is mainly influenced by Insulin-like Growth Factor 1 (IGF-1), in both female and male^36,37^. This factor is primarily produced by the liver and directly promotes proliferation, including at the epiphyseal fugue, causing chondrocytes to proliferate^36,37^. In adolescence, estrogen contributes to the proliferation of the cartilaginous framework in both sexes, and in higher concentrations, it leads to the ossification of the cartilaginous framework^37–39^. In females, higher concentrations of estrogen at an earlier age are responsible for reaching the peak of bone ossification and maturation around the time of menarche (approximately 12 years of age)^38,40,41^. To be concrete, prepubertal girls demonstrate an eight-fold higher estrogen level than same-aged boys^38^. In male, testosterone is converted to estrogen locally, e.g. in the cartilage of the epiphyseal plate, by the aromatase P450 to avoid a systemic effect, even in prenatal development^39,42–44^. In male as well, the cartilage proliferation is directly and ossification indirectly linked to testosterone concentration^39,42^. The rate of mineralization depends on the intermediate enzyme aromatase P450, leading to a prolonged interval of bone mass gaining before reaching estrogen concentrations needed for epiphyseal mineralization^37,39,40,42^. The expression and activity of aromatase are sex-specific, and in later stages of life, in males, it can reach the threefold activity of aromatase found in females^44–47^. Furthermore, estrogen inhibits bone resorption (osteoclasts) and modulates osteoblastic/osteocystic activity to maintain bone formation processes^48–50^. In consequence, the bone maturation and subsequent bone remineralization in both sexes can be initiated and enhanced by externally applied circulating estrogen^48–52^.

Regarding the sex-specific ossification stages of the medial clavicular epiphysis, various studies using similar techniques revealed significant sex-specific differences in stage-specific mean age in different populations, including Germany^1,3,5,53^, France^54^, Turkey^55–57^, Thailand^58^ and Australia^6^. A recent study compared the results with those of previous studies that used similar reading-based evaluation techniques, in particular the method of Wittschieber et al.^57^. They followed a two-step process, initially evaluating the images and reevaluating one week later to assess consistency and reliability^57^. If there was a variation in the developmental status between the left and right clavicle, the side with more advanced development was prioritized for the stage diagnosis^57^. In advanced clavicle stages (Schmeling’s stages 2 and 3, respectively Kellinghaus’ substages 2a to 3c) Ramadan et al. (2017) detected greater differences between male and female than in the early and the late Schmeling’s stages (1, 4 to 5)^57^. These observations were consistent with other studies correlating the sex-stratified clavicle stages to the biological age^53,54,56,57,59^.

Therefore, the relevance of 1) sex-based and 2) intra individual side-dependent differences in clavicle maturation is still insufficient investigated. Furthermore, one can hypothesize that biological sex could have a significant influence on processes of ossification and maturation of body tissue, such as epiphyseal fugue of the medial clavicle, particularly emerging at adolescence with more advanced maturation of the clavicle in women.

To assess the predictive power of sex-weighted age estimation based on established clavicle stages is a new field of investigation. A sufficient comprehensive and in-depth analysis of various clavicle stages with intra-individual comparisons had not been carried out previously. Furthermore, the current methods used for age estimation rely on discontinuous methodological processes. These methods involve assessing the biological age by correlating the most advanced clavicle stage with the evidence-based mean values of age in clavicle stages, neglecting the less developed side. However, in order to improve the accuracy of age estimation, the aim was to develop a continuous function based on the established method proposed by Wittschieber et al^60^. Hence, we investigated the sex-dependant regression analysis of real biological age with degrees of maturation of the clavicle separated for both sides aiming a new reliable method of formula based age calculation using clavicle stages and biological sex.

## Materials and methods

### Study design and protocol

For the current analysis, all CT scans between 2012 and 2022 were evaluated for this retrospective study acquired during the routine care at the University Medical Center Mainz, Germany. The authors guarantee the study was conducted in compliance to the Declaration of Helsinki from 1964 and to the local ethical guidelines. This retrospective study analyzed data obtained during the routine care. Routine care imaging was performed under medical indications and with patients’ informed consent to computed tomography imaging, including the use of the acquired data for scientific purposes. In consequence, the Ethics Committee of the *Landesärztekammer Rheinland-Pfalz* [engl.: “State Medical Association of Rhineland-Palatinate”] approved the study conductance (No.: 2023-16,971). The article follows the STARD guideline of 2015 (Supplemental Material).

This retrospective, single-blinded, monocentric study aimed to determine a continuous arithmetic approximation for the known biological age by using the established ossification stages of the medial clavicle epiphysis and the biological sex. Three readers blinded for the real (or known) age and biological sex determined the clavicle stages, following the categorization system by Schmeling et al. and Kellinghaus et al^3–5^., and subsequently estimated the biological age according to the method of Wittschieber et al. (Table 1)^53,60,61^. The three readers, each with 6 months of experience in determining clavicle stages, evaluated the CT datasets within the same four-week period, between 5:00 and 10:00 p.m. using radiological imaging monitors calibrated according to DIN ISO 6868-157 standards. Reporting was conducted under standardized conditions at the Department of Neuroradiology, following guidelines set by the Swiss Society for Diagnostics^62^. Data were compiled in a separate pseudonymized table and the biological age has been added subsequently for analysis. To avoid an observation bias, two tables were created, including one for the known age and another for the assessed stages of the right and left clavicles.

**Table 1:** Age estimation after the method of Wittschieber et al. using the clavicle ossification stages after Schmeling et al. and Kellinghaus et al.

Clavicle stages^3–5^		Definition^3–5^	Schematic^3–5^	Sex	N	Age after Wittschieber et al^53,60,61^.	
						Range [min–max]	Mean
Stage 1		Ossification center not ossified		Male	12	10.7–14.9	12.9
				Female	4	12.1–15.4	14.3
Stage 2		Ossification center partly ossified. epiphyseal cartilage not ossified (Stage 2a-c)		Male	16	15.0–20.4	17.4
				Female	8	14.1–18.4	16.0
	Substage 2a	Width of the ossification center ≤ 1/3 of the metaphyseal width		Male	4	15.0–16.5	15.7
				Female	4	14.8–18.4	16.2
	Substage 2b	Width of the ossification center between 1/3 and 2/3 of the metaphyseal width		Male	10	16.1–20.4	17.8
				Female	3	14.1–15.8	15.2
	Substage 2c	Width of the ossification center > 2/3 of the metaphyseal width		Male	2	17.4–20.2	18.8
				Female	1	17.9	-
Stage 3		Ossification center ossified. epiphyseal cartilage partly ossified (Stage 3a-c)		Male	108	16.4–36.5	22.2
				Female	53	15.5–23.3	20.7
	Substage 3a	Epiphyseal-metaphyseal fusion ≤ 1/3 of the metaphyseal width		Male	24	16.4–22.3	19.6
				Female	12	15.5–23.3	19.0
	Substage 3b	Epiphyseal-metaphyseal fusion between 1/3 and 2/3 of the metaphyseal width		Male	31	17.6–36.5	21.7
				Female	15	16.4–23.3	19.7
	Substage 3c	Epiphyseal-metaphyseal fusion > 2/3 of the metaphyseal width		Male	53	19.0–30.0	23.6
				Female	26	19.4–26.5	22.0
Stage 4		Epiphyseal cartilage completely ossified *with* visible scar		Male	180	21.6–40.5	29.7
				Female	65	21.1–37.3	27.2
Stage 5		Epiphyseal cartilage completely ossified *without* visible scar		Male	20	26.6–40.0	31.6
				Female	27	26.7–39.6	32.9

### CT scan metrics and inclusion criteria

**Figure 1:**
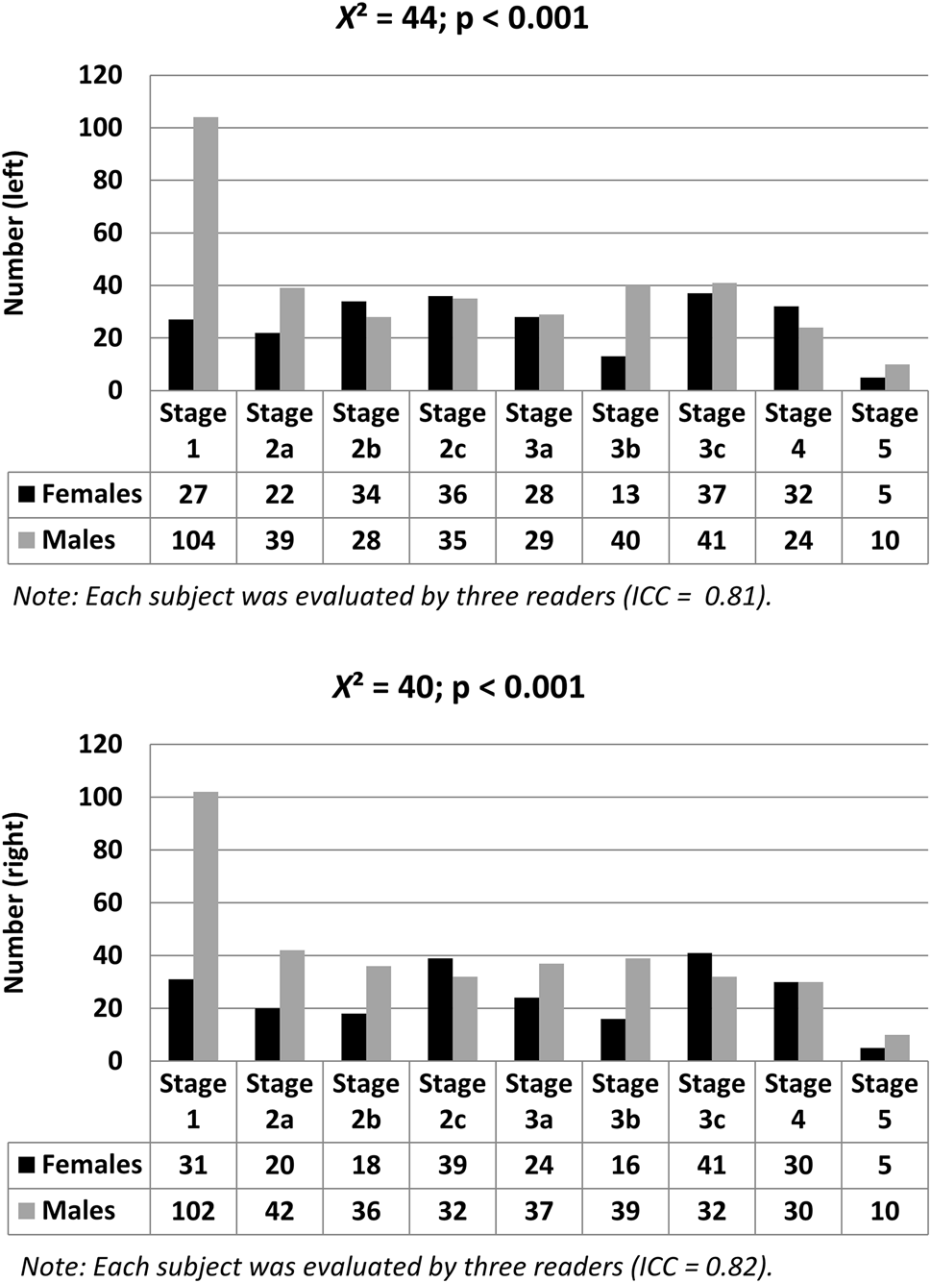
Overview of the number of observed stages in the (**A**) left and (**B**) right clavicle clustered by sex. Mean age in women differed from that in men (18.81 ± 3.46 years vs. 17.72 ± 2.95 years; *p* = 0.02).

CT scans with a standardized slice thickness of 1 mm in 3D multiplanar reconstruction, 120 kV and automatic dose rate control (min. 15 mAS) were included to the study, under the condition of an assessable image quality and the biological age of less or equal than 25 years at the time point of CT image acquisition. It is important to note that the accuracy of age estimation is unaffected by the CT scan dose^63^; however, professional expertise and slice thickness has been identified as a significant factor in this regard^57,60,61^. The condition of an assessable image quality was proofed by a neuroradiologist with more than ten years of experience. Furthermore, subjects with metabolic disorders mentioned in their medical history were excluded from the analysis, however, metabolic alterations could not be completely ruled out. For this purpose, hormone level measurements would have been required. In accordance with the study selection criteria n = 198 data sets were included (n = 78 female; n = 120 male; 12–25 years of biological age; see Fig. 1).

### Process of age estimation and statistics

**Figure 2:**
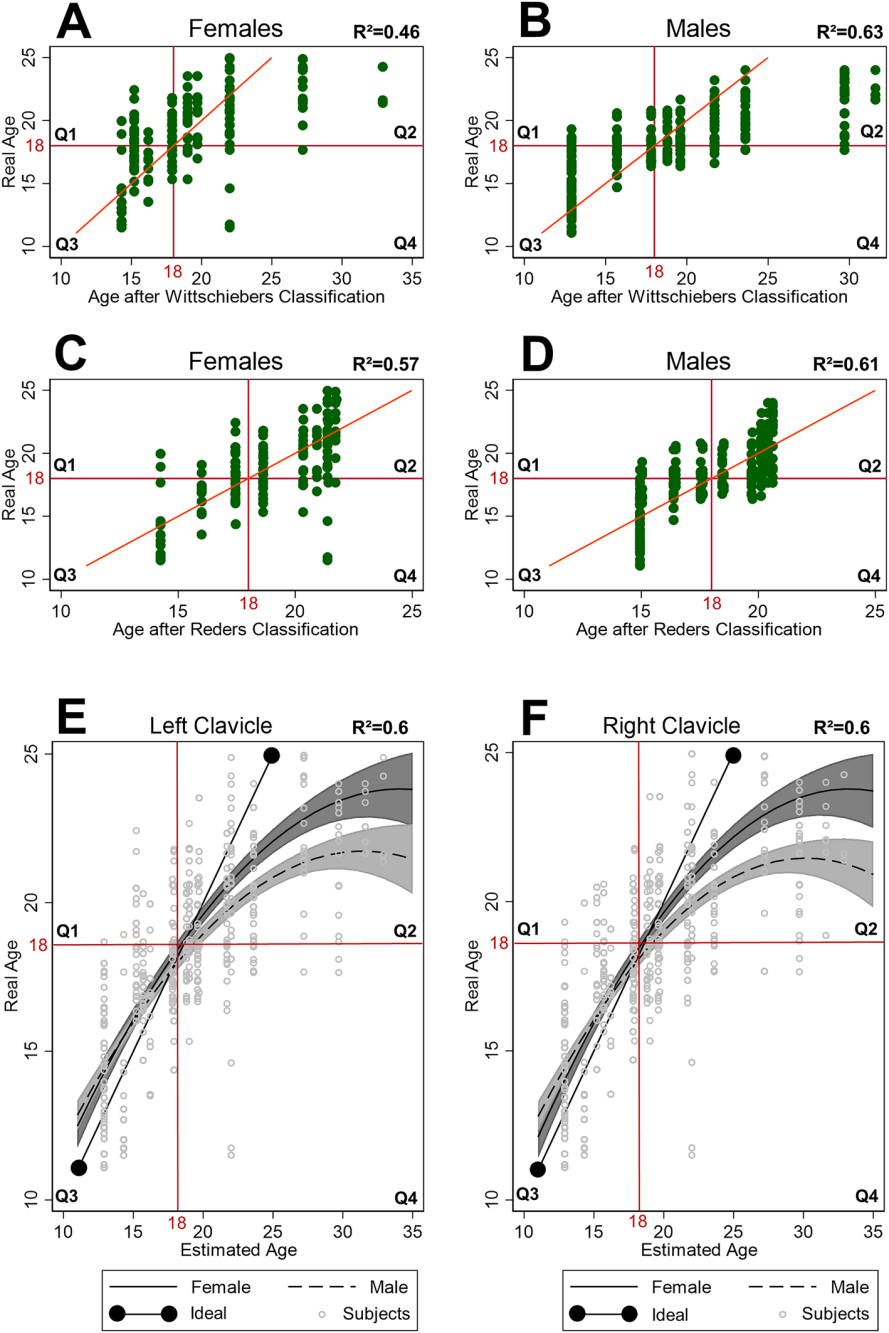
Comparison of Gender-Specific Age Estimation According to Wittschieber et al. and Reder et al. Each subject was assigned specific x- and y-variables (visualized as dots; each individual is represented by three dots in the case of three readers). The x-coordinate was defined by the method-specific estimated age, and the y-coordinate was assigned to the real biological age. The coordinate systems were divided into quadrants (Q1-4). A red line running between quadrants Q2 and Q3 marks the ideal line for a 1:1 correlation. Both a vertical and a horizontal red line represent the critical age of 18 years (vertical: estimated age of 18; horizontal: real age of 18). Subjects located in Q1 are estimated to be under 18, even though they are already at least 18. Those in Q2 and Q3 are estimated approximately at their real age, and subjects located in Q4 are estimated to be over 18, even though they are under 18. The comparison of the method according to Wittschieber et al. (**A** and **B**) with that of Reder et al. (**C** and **D**) shows no significant difference in age estimation in men (**B** vs. **D**), but a clear difference in age estimation in women (**A** vs. **C**). Furthermore, the figures according to Wittschieber et al. refer to the less developed side of the clavicle, whereas the figures according to Reder et al. are applicable to both clavicles of an individual (**E** and **F**).

Intraclass correlation coefficients (ICC; or “Interrater agreement”) over the three readers were ICC = 0.81 (stages of the left clavicle) and ICC = 0.82 (stages of the right clavicle), and, subsequently for 0.75 ≤ ICC ≤ 0.9, assessable as “good”^64–67^. Thus, clavicle stages of n = 78 women and n = 120 men for both sides each were included for calculations. For further evaluation of the established methods and, subsequently, defining a quantitative arithmetical term for age estimation based on the semi-quantitative method of Wittschieber et al^60^., the methodological process was as follows:

I) According to Table 1, determination of the clavicles’ maturation stages after Schmeling et al. and Kellinghaus et al. based on the present CT imaging data^3–5^.
II) Age determination after the established method according to Wittschieber et al.: Correlation of the clavicle stages with the AGFAD reference tables for age determination estimating individuals age after Wittschieber et al^53,60,61^.(see Table 1).
III) Age determination after the method according to Reder et al.: In accordance with the assumed minimum age (clavmin) and the expected linear prediction of the real age, the clavicle stages were recoded: 1 at clavmin = 14 years, 2a at clavmin = 15 years, 2b at clavmin = 16 years, 2c at clavmin = 17 years, 3a at clavmin = 18 years, 3b at clavmin = 19 years, 3c at clavmin = 20 years, 4 at clavmin = 21 years and 5 at clavmin = 22 years. Female sex was coded as '0,' and male as '1'. The known age in years was calculated by subtracting the birthday from the day of examination. It was not truncated to the year but kept with decimals; hence it should be precise about ± 1 day. Ordinary least square regression for the definition of a continuous age estimation function via linear, quadratic and interaction terms was performed. For regression analysis, the biological age was defined as the target parameter to be predicted. Taking into account interactions between clavicle stages of both clavicles (represented by the assumed minimum age = clavmin) in each individual and considering interactions between clavicle stages and biological sex, a regression term was defined (see Supplementary Methods S1 and S2; see Table 2). Only significant terms were kept in the equation (*p* < 0.01). Analyses were calculated by STATA (V16, STATA Corp., Texas, USA). Based hereon, a new reference table was created (Table 3).To visualize, a subsequent dataset was generated including each subject (values for both clavicles evaluated by the three readers), with individual "estimated age" for each method and "known age" defined as x- and y-values on a two-dimensional coordinate system (see Fig. 2). As a result, each subject is represented as 3 dots in the figures (three readers).
IV) According to the power analysis for regression models, a group size of n = 78 in women (n = 120 in men), four predictors, an alpha level of 0.05 (5%), and a recommended power of 0.9, a determination coefficient R^2^ = 0.17 (R^2^ = 0.12 in men) would be necessary to achieve significant effects.

**Table 2:** Clavicle Stage- and Sex-based Regression analysis of Reders Method for age estimation.

Age estimation	R^2^ = 0.6_,_ F_4; 589_ = 216.72, *p* < 0.001					
Variables	Beta	SE_Beta_	t-value	95% CI	99% CI	*p*-value
Clavicle stage	3.97	0.78	5.10	2.44; 5.5	1.95; 5.97	< 0.001
(Clavicle stage)^2^	− 0.08	0.02	− 3.67	− 0.12; − 0.04	− 0.13; − 0.02	< 0.001
Sex	6.13	1.43	4.28	3.32; 8.95	2.43; 9.84	< 0.001
Sex*clavicle stage	− 0.33	0.08	− 4.27	− 0.48; − 0.18	− 0.53; − 0.13	< 0.001
Constant	− 27.86	7.11	− 3.92	− 41.81; − 13.9	− 46.22; − 9.49	< 0.001

The clavicle stages are coded into the values of the assumed minimum age (clavmin; from 14 to 22) and inserted in this form into the term (e.g. Clavicle stage 2b = 16). Women are coded as ‘0,’ and men as ‘1’. The values are provided as regression coefficient Beta, the standard error of Beta (SE_Beta_), and in 95% and 99% confidence intervals of Beta. The term is applicable regardless of the side of the examined clavicle. The values refer to the data output under Supplementary Methods S1 and S2.

**Table 3:** Precision Comparison of the Estimation Methods.

Sex	Wittschiebers method	Reders method			
	N	MAE	RMSE	MAE	RMSE
Overall					
Female	78	2.57	3.19	1.54	1.82
Male	120	2.57	3.47	1.54	2.25
Subjects under 18 years of age					
Female	39	1.96	2.62	1.30	1.41
Male	63	1.96	2.59	1.30	1.57

For the comparison of the precision of both methods, the mean absolute error (MAE) and the root mean squared error (RMSE) were used as quality criteria. The higher the values, the higher the probability of error for the method. Both methods were specifically compared with each other, particularly concerning individuals under the age of 18.

### Ethics approval and consent to participate

The authors guarantee the study was conducted in compliance to the Declaration of Helsinki from 1964 and to the local ethical guidelines. This retrospective study analyzed data obtained during the routine care. Routine care imaging was performed under medical indications and with patients’ informed consent to computed tomography imaging, including the use of the acquired data for scientific purposes. In consequence, the Ethics Committee of the *Landesärztekammer Rheinland-Pfalz* [engl.: “State Medical Association of Rhineland-Palatinate”] approved the conductance of this single-blinded monocentric study (No.: 2023-16,971) in accordance with the legislation of the *Landeskrankenhausgesetz § 37 “Datenschutz bei Forschungsvorhaben”, Abs. 1 bis 5* [State Hospital Act of Rhineland-Palatinate, Sect. 37 “Data Protection for Research Projects”, Paragraph 1 to 5].

## Results

The three readers evaluated the clavicle stages for each side in n = 78 female and n = 120 male subjects (ICC ≥ 0.81 each side; n = 234 female clavicle stages; n = 360 male clavicle stages). According to Fig. 1, within the included range of real biological age from 12 to 25 years, in female subjects the clavicle stage 3c was the most evaluated with n = 78 (left: n = 37; right: n = 41) and stage 2c was the second most observed with n = 75 (left: n = 36; right: n = 39), whereas in male stage 1 was the most observed with n = 206 (left: n = 104; right: n = 102) and stage 2a was the second most evaluated with n = 81 (left: n = 39; right: n = 42). In mean, the real/known biological age in women was 18.81 ± 3.46 years and in men 17.72 ± 2.95 years (*p* = 0.02).

The ordinary least square regression revealed four relevant predictors (or variables) for the approximation of the real (or known) age by using established clavicle stages, which has been recoded into their assumed minimum age (clavmin) (see 2.3; Table 2; Supplementary Methods S1 and S2):

I. Clavicle Stage: coded continuously from 14 to 22 for each clavicle stage: 1 = 14; 2a = 15; 2b = 16; 2c = 17; 3a = 18; 3b = 19; 3c = 20; 4 = 21 and 5 = 22 (*p* < 0.001 each side).
II. Clavicle Stage^2^: Quadratic clavicle stage coded into clavmin from 14 to 22 (*p* < 0.001 each side).
III. Sex: Biological sex, whereas the female sex was defined as “0” and the male as “1” (left: *p* = 0.021; right:* p* = 0.001).
IV. Clavicle Stage*Sex: Interaction term of clavicle stage (represented by the clavmin) and sex, the product of the multiplication from the clavmin (14 to 22) by the biological sex (or: clavicle stage*sex; *p* < 0.001 each side).

The analysis revealed a strong predictive power for estimating the real biological age based on both the left and right clavicle (R^2^ = 0.6 and p < 0.001 each side; F_both_ (4; 579) = 216.72; see Table 2; see Supplementary Methods S1 and S2). According to Cohen’s lowest estimation (1988), for R^2^ > 0.26 there is a high variance elucidation^68^. Therefore, no relevant side-dependent altered approximation was observable. By using the regression term, the mean absolute error (MAE) and root mean squared error (RMSE) in the age estimation could be reduced for women (MAE from 2.57 to 1.54 and RMSE from 3.19 to 1.82), and for men (MAE from 2.57 to 1.54 and RMSE from 3.47 to 2.25), as to compare with Table 3.

Hence, quadratic regression functions are calculable by using the variable-specific Beta (Table 2) and the recoded clavicle stages (see 2.3; Table 1). The results were compiled within the framework of a reference table (Table 4).

**Table 4:** Reference table of estimated sex- and clavicle stage-based ages after the method of Reder et al.

s	Sex	Mean age	SE	t-value	Age range (95% CI)	Age range (99% CI)
1	Female	12.51	0.42	29.66	11.68; 13.34	11.42; 13.6
	Male	14.0	0.24	58.67	13.54; 14.47	13.39; 14.62
2a	Female	14.24	0.28	50.6	13.69; 14.79	13.51; 14.96
	Male	15.4	0.15	102.8	15.1; 15.69	15.01; 15.78
2b	Female	15.81	0.2	78.71	15.41; 16.2	15.29; 16.33
	Male	16.64	0.14	116.66	16.35; 16.91	16.27; 17.0
2c	Female	17.22	0.18	98.38	16.88; 17.56	16.77; 17.67
	Male	17.72	0.17	107.68	17.39; 18.04	17.3; 18.14
3a	Female	18.48	0.17	106.91	18.14; 18.82	18.03; 18.93
	Male	18.65	0.17	108.18	18.31; 18.99	18.2; 19.09
3b	Female	19.59	0.17	116.05	19.26; 19.92	19.15; 20.02
	Male	19.42	0.16	120.6	19.11; 19.74	19.01; 19.84
3c	Female	20.53	0.17	123.78	20.21; 20.86	20.11; 20.97
	Male	20.04	0.16	129.13	19.74; 20.35	19.64; 20.44
4	Female	21.33	0.2	108.4	20.95; 21.72	20.83; 21.84
	Male	20.51	0.21	98.7	20.1; 20.91	19.97; 21.04
5	Female	21.98	0.3	75.6	21.41; 22.55	21.23; 22.73
	Male	20.82	0.34	61.97	20.16; 21.48	19.95; 21.68

The established clavicle stages according to Schmeling and Kellinghaus, biological sex, the mean observed age, standard error (SE), t-value, and age range in years for the 95% and 99% confidence intervals are reported. The values refer to the data output under Supplementary Methods S1 and S2.

In Fig. 2, the correlation between the estimated age after Wittschieber et al. (A and B) and the estimated age based on quadratic functions of sex-dependent estimation of the real biological age were depicted for female and male (C and D). The plots of the subjects are divided into quadrants (Q1–Q4) and intersected by a red line running from Q3 to Q2. This line is the ideal line. Subjects located in Q1 are estimated to be under 18, even though they are already at least 18. Those in Q2 and Q3 are estimated approximately at their real age, and subjects located in Q4 are estimated to be over 18, even though they are under 18. In Fig. 2 E and F, the 95% CI and the mean age estimated according to the method after Reder et al. was visualized for the left and right clavicle in separate. The light gray and centrally dashed curve refers to the age estimation in women, whereas the dark gray and centrally solid curve relates to men. From the curve patterns, it is apparent that age estimation closely aligns with the ideal line until approximately 18 years. However, after this age, the curves for women and men intersect and diverge significantly, spreading apart until the real age of approximately 21 years and the curves flattens significantly in their maximum slope. In reverse, this implies that age is underestimated after 21 years for both sexes. Notably, women exhibit more advanced clavicle stages compared to men at the same biological age (higher estimated age means more advanced clavicle stages). Furthermore, in some cases, extreme values were observed. For instance, despite the known age being below 13 years, the estimated age based on clavicle stages was determined to be over 20 years. Conversely, there were instances of reverse extremes: In those cases individuals with a real biological age well over 23 years were classified with less advanced clavicle stages and consequently estimated age of around 16 years.

The Wittschieber method demonstrated a similar quality of age prediction in male subjects (R^2^ approximately 0.6 in both methods; Fig. 2A–D), however, these results refer to the less developed clavicle side regarding Wittschieber et al., and to both clavicle sides regarding Reder et al. (Fig. 2E and F). In female subjects, the prediction accuracy was significantly improved by the method of Reder et al. (R^2^ increased from 0.46 to 0.57; Fig. 2A–D). The error values (MAE and RSME) in age estimation decreased with the use of the method after Reder et al. for the entire population and for those with a real age under 18 years (see Table 3).

## Discussion

The medial clavicular epiphysis is well-established in age estimation as one of the last bones to ossify^2,3^. Moreover, it is widely acknowledged in scientific literature as a reliable biological structure for forensic age estimation with a crucial role in determining legal adulthood^4,5,53,57,59,60^. Its development is dividable into nine stages (Schmeling et al. and Kellinghaus et al.), with stage 3c defined as the completion of the 19th year of life^1,3–5,53,57,59,60^. There were no significant sex-specific differences observed in the earlier stages of ossification of the medial clavicular epiphyseal fusion up to an estimated age of approximately 16 years (or stage 2a)^5,53,57,59^. However, in the later stages (approximately correlated with 20 years of age), in the most of the previous studies in females earlier maturation stages were observable compared to males^4,5,53,57,59^. Considering that age estimation is based on discontinuous methods (assigning age mean values to clavicle stages in a standardized manner by numerous authors), we aimed to develop continuous arithmetic functions based on the established method after Wittschieber et al. for the possibility of further evaluation^60^, consideringno information was available about the precision of the age estimates via clavicle—particularly regarding left and right clavicle and women and men separately. Additionally, previous age estimation did consider the age of left and right clavicle, but only by taking the lower value of both sides. In the present article, we do the same in the present revision but consider additionally neighbour stages. This is particularly relevant in small groups of certain developmental stages. In the majority of cases, the curve patterns of our results revealed a close correlation between the estimated and real age until approximately 18 years of age, comparable to previous studies^6,54–57^. However, beyond this age, the function for age estimation in women and men began to diverge significantly, indicating that the real age could be overestimated for both sexes by using the established methods. It is noteworthy that women demonstrated more advanced clavicle stages compared to men at the same biological age, subsequently estimating a higher biological age due to more advanced clavicle stages. This would align with the observations of many studies that have compared sex hormone levels with bone age and cartilage tissue mineralization^37–44^. Women tend to have an earlier onset and achieve higher cartilage mineralization levels than men^38,40,41^. In men, there is an initial delay in bone ossification in prepubertal ages, appearing to be due to the involvement of the aromatase P450, transforming testosterone to estrogen^37,39,40,42–44^. This results in an extended phase of cartilage proliferation and exceeds in a higher bone mass after mineralization^37,39,40,42^. The puberty, this delay in ossification is quickly compensated for, as the expression and activity of the aromatase P450 can significantly increase^44–47^. In individuals over 18 years of age, the curve estimation (Fig. 2 E and F) in women closely follows the ideal line. In men, it flattens significantly. This could be attributed to the fact that men enter puberty later, and bone maturation is regulated through the intermediate step of aromatase^44–47^. Different aromatase activity could lead to greater divergence in the maturation of male clavicles until the age of 25^44–47^. However, in case of the greater disparities from approximately 18 years of age it can be assumed that another influential factor could play a significant role in the maturation of the clavicle, not captured in our study. In this case, the blood levels of sex hormones or environmental factors would be of interest^4,10–16,29,30^. Regarding both age estimation methods, there appears to be a notable tendency to overestimate bone age, particularly in isolated cases involving women. In comparing Fig. 2, the bone age of a 12-year-old subject was estimated to be approximately 21 years. Such overestimations could be detrimental in forensic age determination. It is crucial to consider supplementary methods for age determination in these cases. One such method involves comparing the ossification stage with the actual height and other parameters. If there is a discrepancy between the height (very short or tall stature) and the mental development, it could indicate an unknown hormone-related underlying condition. Premature pubarche or adrenarche is one such condition to mention, affecting girls at an incidence of 1 in 100 children, with a ratio of 5:1 in favor of girls^69,70^. Those affected are physically advanced and typically overweight, in contrast to their cognitive developmental stage. In laboratory tests, a significantly elevated level of dehydroepiandrosterone sulfate (DHEAS) and free testosterone would be noticeable^71,72^. In these girls, the bone age would, on average, correspond to an estimated age more than 2 years above their actual age^71,73^. On the other hand, it is possible that due to the significantly increased use of contraceptives at a young age, bone mineralization could be accelerated^52,74,75^. In this case, a laboratory check of hormones in the hypothalamus-pituitary–gonadal axis and a correlation with medical history would be advisable. To enhance the precision of age determination based on anatomical features, other entities such as dental development can be considered^76^. In the context of anatomy-based age assessment for legal purposes, hence, there is a risk of misclassifying younger individuals as older and thus holding them criminally liable, while older individuals may be misclassified as underage or immature. In case of justified doubts (e.g., cognitive immaturity), it is crucial to conduct urgent additional assessments, such as determining cognitive maturity and blood (sex) hormone levels. Furthermore, it is advisable to apply age estimation based on the lower limit of the 99% confidence interval according to Reder et al. (Table 4) in doubtful cases.

There are several limitations that need to be addressed. First, the variability in CT scan dose based on the automated dose rate control should be mentioned. Nevertheless, it should be noted that the accuracy of age estimation is not influenced by the CT scan dose^63^. However, factors such as professional expertise and the thickness of the scan slices have been identified as significant considerations in this context^57,60,61^. To minimize these influences, (1) the assessment of image quality was conducted by a neuroradiologist with more than 10 years of experience and (2) three readers with comparable experience in evaluating clavicle stages analyzed the image data using multiplanar 3D reconstructions with a standardized slice thickness of 1 mm. The clavicle stage based “estimated age” was correlated to the specific highest observed age (after Wittschieber et al.), as it is commonly practiced. However, the low experience of the readers in clavicle-based age estimation (< 1 year) might have led to extreme outliers in age estimation. This could be partially compensated by including the estimated ages of n = 198 subjects with known age between 12 and 25 years from each of the three readers in the calculations (n = 589 overall). This compensatory effect was reflected in the good interclass correlation coefficient (ICC) and the high determination coefficient regarding to the quadratic function based estimation of the real biological age (R^2^ > 0.6). Regarding overfitting, ordinary regression models exhibit lower susceptibility, however, the inclusion of quadratic terms heightens the risk. Various studies have delved into this phenomenon^77–79^. The most widely accepted view suggests that a ratio of cases to the number of predictors of approximately 13–15 observations (number of subjects per number of predictors) is deemed "reasonably good”^77–79^. In this study, we have 589 subjects with 4 predictors, resulting in a ratio of approximately 147. Overfitting is not entirely ruled out by this, but the impact is assumed to be minimal. In the case of outliers, the quadratic function was able to compensate for erroneously overestimated age and approximate the estimated age to the real age. Conversely, older individuals might be inaccurately classified as underage or immature. Furthermore, within the scope of the retrospective study design and due to potentially incomplete medical anamnesis, the presence of disorders influencing ossification processes, such as premature adrenarche, could not be completely ruled out. In cases of legitimate doubts (e.g., cognitive immaturity), it is imperative to promptly conduct supplementary assessments, encompassing the evaluation of cognitive maturity and blood (sex) hormone levels. The precision of age determination through anatomical features could be improved involving additional factors like dental development^76^. Despite these uncertainties, our quadratic function was able to explain a large amount of variances.

To conclude, a accurate calculation method was evaluated and compared to established techniques for age estimation, correcting for sex and addressing cases of erroneously over- or underestimated age. This allows for subsequent analysis to determine further variables and to quantify their influence on the clavicle maturation. On the one hand, it became clear that the established methods estimate the biological age well up to 18 years. Beyond this, the biological age is increasingly overestimated. On the other hand, sex-specific differences in the maturation of the medial clavicle epiphyseal fugue become particularly evident from approximately 18 years of age onwards, whereas in women, the clavicle matures earlier compared to men. Prior to the age of 18 years, minor sex-based differences were observable. Nevertheless, there were isolated cases of overestimations of the real age. If there are reasonable concerns, such as cognitive immaturity not aligning with anatomical maturity, the lower limit of the 99% confidence interval according to the reference table after Reder et al. should be used for estimating real biological age. Furthermore, supplementary assessments should be taken into account, including the evaluation of blood (sex) hormone levels, the evaluation of the tooth development, and a neuropsychiatric assessment.

### Supplementary Information

Supplementary Information.

## Data Availability

Regarding to the anatomical aspects of the used image data and the possibility to reconstruct facial structures, data is available at the Department of Neuroradiology at the University Medical Mainz. Inquiries have to be send to the director (M.A. Brockmann, MD, MSc). Each request should be based on a scientific hypothesis and reviewed by a (local) ethical committee. Any request must be made in writing. Data will be saved for ten years after publishing (according to Good-Clinical-Practice-guidelines).
