# Supplementary material for: Comparing a common clavicle maturation-based age estimation method to ordinary regression analyses with quadratic and sex-specific interaction terms in adolescents
Source: Sci Rep. 2024 Feb 2;14:2754. doi: 10.1038/s41598-024-52980-x (PMC10837444; doi:10.1038/s41598-024-52980-x)
Supplement: Supplementary file 1 — Supplementary Information. [file 41598_2024_52980_MOESM1_ESM.docx]

Supplementary Methods S1: STATA data output for values of the 95% confidence interval using quadratic and interaction terms in a regression analysis.

reg age c.clavmin##Gender c.clavmin##c.clavmin, 1 (95)
note: clavmin omitted because of collinearity.

      Source |       SS           df       MS      Number of obs   =       584
-------------+----------------------------------   F(4, 579)       =    216.72
       Model |   3501.9275         4  875.481874   Prob > F        =    0.0000
    Residual |  2338.99396       579  4.03971322   R-squared       =    0.5996
-------------+----------------------------------   Adj R-squared   =    0.5968
       Total |  5840.92145       583  10.0187332   Root MSE        =    2.0099

-------------------------------------------------------------------------------------
                age | Coefficient  Std. err.      t    P>|t|     [95% conf. interval]
--------------------+----------------------------------------------------------------
            clavmin |   3.965575   .7772928     5.10   0.000     2.438918    5.492232
                    |
             Gender |
              Male  |   6.135192   1.432272     4.28   0.000     3.322109    8.948274
                    |
   Gender#c.clavmin |
              Male  |  -.3316444    .077759    -4.27   0.000    -.4843685   -.1789202
                    |
            clavmin |          0  (omitted)
                    |
c.clavmin#c.clavmin |  -.0772907   .0210641    -3.67   0.000     -.118662   -.0359194
                    |
              _cons |   -27.8571   7.105727    -3.92   0.000    -41.81324   -13.90096
-------------------------------------------------------------------------------------

. margins Gender, at(clavmin=(14(1)22))

Adjusted predictions                                       Number of obs = 584
Model VCE: OLS

Expression: Linear prediction, predict()
1._at: clavmin = 14
2._at: clavmin = 15
3._at: clavmin = 16
4._at: clavmin = 17
5._at: clavmin = 18
6._at: clavmin = 19
7._at: clavmin = 20
8._at: clavmin = 21
9._at: clavmin = 22

------------------------------------------------------------------------------
             |            Delta-method
             |     Margin   std. err.      t    P>|t|     [95% conf. interval]
-------------+----------------------------------------------------------------
  _at#Gender |
   1#Female  |   12.51197   .4218945    29.66   0.000     11.68334     13.3406
     1#Male  |   14.00414   .2386937    58.67   0.000     13.53533    14.47295
   2#Female  |   14.23611   .2813449    50.60   0.000     13.68353    14.78869
     2#Male  |   15.39664   .1497689   102.80   0.000     15.10248     15.6908
   3#Female  |   15.80568   .2008111    78.71   0.000     15.41127    16.20008
     3#Male  |   16.63456   .1425863   116.66   0.000     16.35451    16.91461
   4#Female  |   17.22066   .1750423    98.38   0.000     16.87686    17.56445
     4#Male  |   17.71789   .1645425   107.68   0.000     17.39472    18.04107
   5#Female  |   18.48106   .1728647   106.91   0.000     18.14154    18.82057
     5#Male  |   18.64665   .1723619   108.18   0.000     18.30812    18.98518
   6#Female  |   19.58687   .1687724   116.05   0.000     19.25539    19.91836
     6#Male  |   19.42082   .1610296   120.60   0.000     19.10455     19.7371
   7#Female  |   20.53811   .1659097   123.79   0.000     20.21225    20.86397
     7#Male  |   20.04042   .1551937   129.13   0.000      19.7356    20.34523
   8#Female  |   21.33477   .1968174   108.40   0.000      20.9482    21.72133
     8#Male  |   20.50543   .2077646    98.70   0.000     20.09736    20.91349
   9#Female  |   21.97684   .2906804    75.60   0.000     21.40592    22.54776
     9#Male  |   20.81586   .3358812    61.97   0.000     20.15616    21.47555
------------------------------------------------------------------------------

Supplementary Methods S2: STATA data output for values of the 99% confidence interval using quadratic and interaction terms in a regression analysis.

reg age c.clavmin##Gender c.clavmin##c.clavmin, l(99)
note: clavmin omitted because of collinearity.

      Source |       SS           df       MS      Number of obs   =       584
-------------+----------------------------------   F(4, 579)       =    216.72
       Model |   3501.9275         4  875.481874   Prob > F        =    0.0000
    Residual |  2338.99396       579  4.03971322   R-squared       =    0.5996
-------------+----------------------------------   Adj R-squared   =    0.5968
       Total |  5840.92145       583  10.0187332   Root MSE        =    2.0099

-------------------------------------------------------------------------------------
                age | Coefficient  Std. err.      t    P>|t|     [99% conf. interval]
--------------------+----------------------------------------------------------------
            clavmin |   3.965575   .7772928     5.10   0.000     1.956781     5.97437
                    |
             Gender |
              Male  |   6.135192   1.432272     4.28   0.000     2.433703     9.83668
                    |
   Gender#c.clavmin |
              Male  |  -.3316444    .077759    -4.27   0.000    -.5326007    -.130688
                    |
            clavmin |          0  (omitted)
                    |
c.clavmin#c.clavmin |  -.0772907   .0210641    -3.67   0.000    -.1317276   -.0228539
                    |
              _cons |   -27.8571   7.105727    -3.92   0.000    -46.22077   -9.493435
-------------------------------------------------------------------------------------

. margins Gender, at(clavmin=(14(1)22)) l(99)

Adjusted predictions                                       Number of obs = 584
Model VCE: OLS

Expression: Linear prediction, predict()
1._at: clavmin = 14
2._at: clavmin = 15
3._at: clavmin = 16
4._at: clavmin = 17
5._at: clavmin = 18
6._at: clavmin = 19
7._at: clavmin = 20
8._at: clavmin = 21
9._at: clavmin = 22

------------------------------------------------------------------------------
             |            Delta-method
             |     Margin   std. err.      t    P>|t|     [99% conf. interval]
-------------+----------------------------------------------------------------
  _at#Gender |
   1#Female  |   12.51197   .4218945    29.66   0.000     11.42165    13.60229
     1#Male  |   14.00414   .2386937    58.67   0.000     13.38727    14.62101
   2#Female  |   14.23611   .2813449    50.60   0.000     13.50902    14.96321
     2#Male  |   15.39664   .1497689   102.80   0.000     15.00958    15.78369
   3#Female  |   15.80568   .2008111    78.71   0.000     15.28671    16.32464
     3#Male  |   16.63456   .1425863   116.66   0.000     16.26606    17.00305
   4#Female  |   17.22066   .1750423    98.38   0.000     16.76829    17.67303
     4#Male  |   17.71789   .1645425   107.68   0.000     17.29266    18.14313
   5#Female  |   18.48106   .1728647   106.91   0.000     18.03431     18.9278
     5#Male  |   18.64665   .1723619   108.18   0.000     18.20121    19.09209
   6#Female  |   19.58687   .1687724   116.05   0.000     19.15071    20.02304
     6#Male  |   19.42082   .1610296   120.60   0.000     19.00467    19.83698
   7#Female  |   20.53811   .1659097   123.79   0.000     20.10934    20.96688
     7#Male  |   20.04042   .1551937   129.13   0.000     19.63934    20.44149
   8#Female  |   21.33477   .1968174   108.40   0.000     20.82612    21.84341
     8#Male  |   20.50543   .2077646    98.70   0.000     19.96849    21.04236
   9#Female  |   21.97684   .2906804    75.60   0.000     21.22562    22.72806
     9#Male  |   20.81586   .3358812    61.97   0.000     19.94782    21.68389
------------------------------------------------------------------------------
